# Global oral cholera vaccine use, 2013–2018

**DOI:** 10.1016/j.vaccine.2019.08.086

**Published:** 2019-09-10

**Authors:** Lorenzo Pezzoli

**Affiliations:** Cholera Team/Focal Point for Vaccination, Infectious Hazard Management (IHM), World Health Organization, Switzerland

**Keywords:** Oral Cholera Vaccine, Vaccination Campaigns, Global Task Force on Cholera Control, Cholera Elimination, End Cholera

## Abstract

Vaccination is a key intervention to prevent and control cholera in conjunction with water, sanitation and hygiene activities. An oral cholera vaccine (OCV) stockpile was established by the World Health Organization (WHO) in 2013. We reviewed its use from July 2013 to all of 2018 in order to assess its role in cholera control. We computed information related to OCV deployments and campaigns conducted including setting, target population, timelines, delivery strategy, reported adverse events, coverage achieved, and costs.

In 2013–2018, a total of 83,509,941 OCV doses have been requested by 24 countries, of which 55,409,160 were approved and 36,066,010 eventually shipped in 83 deployments, resulting in 104 vaccination campaigns in 22 countries. OCVs had in general high uptake (mean administrative coverage 1st dose campaign at 90.3%; 2nd dose campaign at 88.2%; mean survey-estimated two-dose coverage at 69.9%, at least one dose at 84.6%) No serious adverse events were reported. Campaigns were organized quickly (five days median duration). In emergency settings, the longest delay was from the occurrence of the emergency to requesting OCV (median: 26 days). The mean cost of administering one dose of vaccine was 2.98 USD.

The OCV stockpile is an important public health resource. OCVs were generally well accepted by the population and their use demonstrated to be safe and feasible in all settings. OCV was an inexpensive intervention, although timing was a limiting factor for emergency use. The dynamic created by the establishment of the OCV stockpile has played a role in the increased use of the vaccine by setting in motion a virtuous cycle by which better monitoring and evaluation leads to better campaign organization, better cholera control, and more requests being generated. Further work is needed to improve timeliness of response and contextualize strategies for OCV delivery in the various settings.

## 1. Introduction

*Vibrio cholerae* (serotypes O1 and, to a much lesser extent, O139) is a highly transmissible bacterium, which can cause a rapidly dehydrating, watery diarrheal disease known as cholera [1]. In 2017, 34 countries reported a total of 1,227,391 cases including 5654 deaths to the World Health Organization (WHO), resulting in an overall case fatality rate (CFR) of 0.5% [2]. However, official figures are significantly underreported and the global cholera burden was estimated at 2.86 million cases (range: 1.3–4.0 m) and 95,000 deaths (range: 21,000–143,000) per year and a population at risk of approximately 1.3 billion in 69 endemic countries [3]. Cholera outbreaks are frequent and prolonged in endemic areas with recurrent seasonal patterns [4]. Outbreaks also occur in non-endemic areas, initiated by exogenous introduction of *V. cholerae*, often associated with complex emergencies that result in the breakdown of infrastructure or population displacement [5]. Although cholera affects any age group, children under five years of age are at higher risk of contracting cholera in endemic settings [6].

Successful control of cholera is directly related to improvements in hygiene and availability of clean drinking water as well as sanitary disposal of waste, disease detection and case management, as was seen with the curbing of the Latin American cholera epidemic in the 1990s [7]. It is therefore not surprising that cholera remains a persistent problem in many resource-limited settings where poverty, political instability, natural calamities, or security conditions make implementation of appropriate surveillance and control measures challenging [8]. In those contexts, vaccination can serve as a complementary strategy for cholera prevention and control, which can be implemented for short to medium term, while access to other primary prevention measures such as safe water, sanitation and hygiene (WASH) improves [9]. Oral cholera vaccines (OCVs) currently available in the global market include Dukoral (SBL Vaccin, Sweden), Shanchol (Shantha Biotechnics Ltd, India), and Euvichol (Eubiologics, South Korea) [10,11]. They have an average two-dose efficacy of 58% (95% confidence interval [CI], 42–69%) and effectiveness of 76% (95% CI, 62–85%) for at least 3 years [12], with one study showing efficacy for up to 5 years[13]. Although OCV currently used in mass campaigns are administered according to a two-dose regimen 14 days apart, a single dose provides short-term protection, with a pooled effectiveness of 69% (95% CI 35–85%) within the first year, which has important implications for outbreak management [12].

In 2013, WHO, with funding (i.e. vaccine costs and since 2016 operational costs for campaign implementation) from GAVI, the Vaccine Alliance, created an OCV stockpile to respond to emergency situations [14], including outbreaks and humanitarian crises [15-17]. The OCV stockpile is also used in non-emergency settings as one of the key strategies to contribute controlling cholera in endemic areas (i.e. “cholera hotspots”), [18,19]. Ideally, OCV should be used in conjunction with other preventative measures such as WASH interventions and social mobilization. The global stockpile only includes prequalified vaccines (i.e. meeting WHO recommendations in terms of quality, safety and efficacy) [20]. Prior to 2011, Dukoral was the only WHO prequalified OCV, but since it requires a buffer to be dissolved in potable water prior to administration, its use was not ideal for mass vaccination campaigns [11]. Shanchol (derived from Dukoral thanks to a successful technology transfer agreement, prequalified in 2011) and Euvichol (result of a similar technology transfer and prequalified in 2015) are modified versions of Dukoral, which do not require a buffer, making them more suitable for use during mass vaccination campaigns [10]. These two more recently prequalified OCVs are the vaccines available through the stockpile for public health purposes for all individuals above one year of age living in the targeted areas; whereas Dukoral is predominantly used for travelers. In all settings, a series of criteria should be considered to guide the decision to vaccinate, namely the risk of cholera among the targeted populations and the risk of geographic spread; and the programmatic capacity to cover as many person as possible who are eligible to receive the vaccine and living in the targeted area (e.g. those aged ≥1 year) [9]. Because of constraints on global supply and availability, OCV doses for emergency use are released from the stockpile after review of the requests by the International Coordinating Group (ICG), composed of UNICEF, Médecins Sans Frontières (MSF), the International Federation of Red Cross and Red Crescent Societies (IFRC), and WHO of applications by countries (i.e. primarily Ministries of Health, often with the support of partner institutions) [21]. Since 2016, a mechanism to access OCV in non-emergency settings to contribute controlling endemic cholera in hotspots was established under the OCV Working Group (WG) of the Global Task Force on Cholera Control (GTFCC), a WHO collaborative mechanism between institutions active in cholera control [22]. Non-emergency requests are assessed by the GTFCC OCV WG based on the risk of cholera and contextual criteria (e.g. vulnerabilities which make cholera a recurrent problem), and are conditional on the country’s capacity and commitment for long-term cholera control/elimination. Multisectoral plans integrating the use of OCV with other interventions (most notably WASH improvement plans) are required to be appended to GTFCC requests and are assessed also by WASH specialists within the GTFCC. In general ICG requests result in one vaccination campaign conducted in urgency for a specific emergency; while GTFCC requests tend to be larger and are intended for systematic OCV use as part of a multisectoral National Cholera Elimination or Control Plan (NCP) targeting a country’s cholera hotspots with multiple campaigns which can be rolled out in phases for more than one year [23]. Once approved, shipment of vaccines to requesting countries is handled by UNICEF Supply Division. A minimum quantity (set at two million doses for 2018, and revised yearly based on global supply) is always reserved for emergency use, and the remaining doses are allocated to non-emergency situations depending on availability at any given time [24].

To draw lessons from OCV use in different contexts and improve its implementation, we reviewed information related to OCV deployments and related campaigns conducted since the creation of the stockpile.

## 2. Methods

### 2.1. Study design

We obtained information on OCV doses requested, approved, and shipped globally from July 2013 (date of stockpile creation) until all of 2018 by the ICG or GTFCC secretariats. We obtained information on population targeted, interval from request to delivery (especially with regards to outbreak response), delivery strategy used, adverse events recorded, coverage achieved, and costs incurred from reports of vaccination campaigns submitted by requestors following campaign implementation. We supplemented this information with published reports by using the search terms “cholera vaccination” and “cholera vaccination campaign” on PubMed. We excluded use of OCV from the stockpile related to research studies.

### 2.2. Definitions and measures

OCV doses were requested from the stockpile using standard forms available on the WHO Cholera webpages, either through the GTFCC OCV WG or the ICG. One of these groups either approved or did not approve the request. Doses shipped (i.e. deployments) were the ones eventually deployed to requesting countries, prioritized based on vaccines availability in the stockpile, and whether requests were made in emergency (i.e. outbreak response to curb spread of cholera or humanitarian crisis to prevent a cholera outbreak in a vulnerable setting) or non-emergency (i.e. to contribute controlling endemic cholera in hotspots) settings. A deployment may have resulted in more than one campaign (e.g. doses shipped to cover multiple areas at different times), while a campaign may be the result of more than one deployment (e.g. when doses for the first round and for the second round are shipped separately). The target population was defined as the number of individuals eligible for the vaccine (i.e. above one year of age) who are members of the circumscribed population (e.g. a geographic area) to whom OCV was to be offered. This figure was generally an estimation based on existing administrative population figures, or a more precise figure based on a study census. Administrative coverage was defined as the proportion of the target population who received one dose of the vaccine, during the vaccination round, by dividing the number of doses administered per round (i.e. 1st dose campaign or 2nd dose campaign) by the target population. The estimated vaccine coverage, assessed during population surveys conducted in the close follow-up of the vaccine campaigns, was defined as the percentage of the target population who received at least one or two doses of the vaccine. Adverse events following immunization (AEFI) were defined as reported medical incidents that take place after vaccination and cause concern. An AEFI was considered serious if it resulted in death, was life-threatening, required in-patient hospitalization or prolongation of existing hospitalization, resulted in persistent or significant disability/incapacity, was a congenital anomaly/birth defect, or required intervention to prevent permanent impairment or damage. To allow comparison of the expenses for vaccination across various campaigns, the costing lines were categorized: cost of vaccines, international shipment, and operational costs. The delivery cost per fully immunized person was calculated using the total local expenses incurred (excluding vaccine, international shipment and technical support costs) as the numerator and the number of fully immunized persons as the denominator.

Key dates of OCV campaign events were taken from various sources including outbreak situation reports (sitreps), campaign reports, and ICG / GTFCC secretariats’ records. Major milestones included in the timeline for OCV vaccination were as follows: (1) planning events (from the date of the first laboratory confirmation of cholera case to the dates of the official decision to use OCV and of the request; (2) administrative events (from receipt of OCV request, to approval and eventual arrival of shipment to the country); and (3) vaccine implementation events (for both rounds of vaccination, when applicable). For emergency vaccination, time to partial protection was defined as the interval from laboratory confirmation of cholera or the occurrence of the humanitarian crisis to seven days after start of the first round, in days. Time to full protection was defined as the interval from laboratory confirmation to seven days after end of the second round, in days. We obtained data on OCV use before the creation of the stockpile (pre-2013) starting from 1997 from a previous publication [25].

## 3. Results

### 3.1. Deployments

**Fig. 1.**
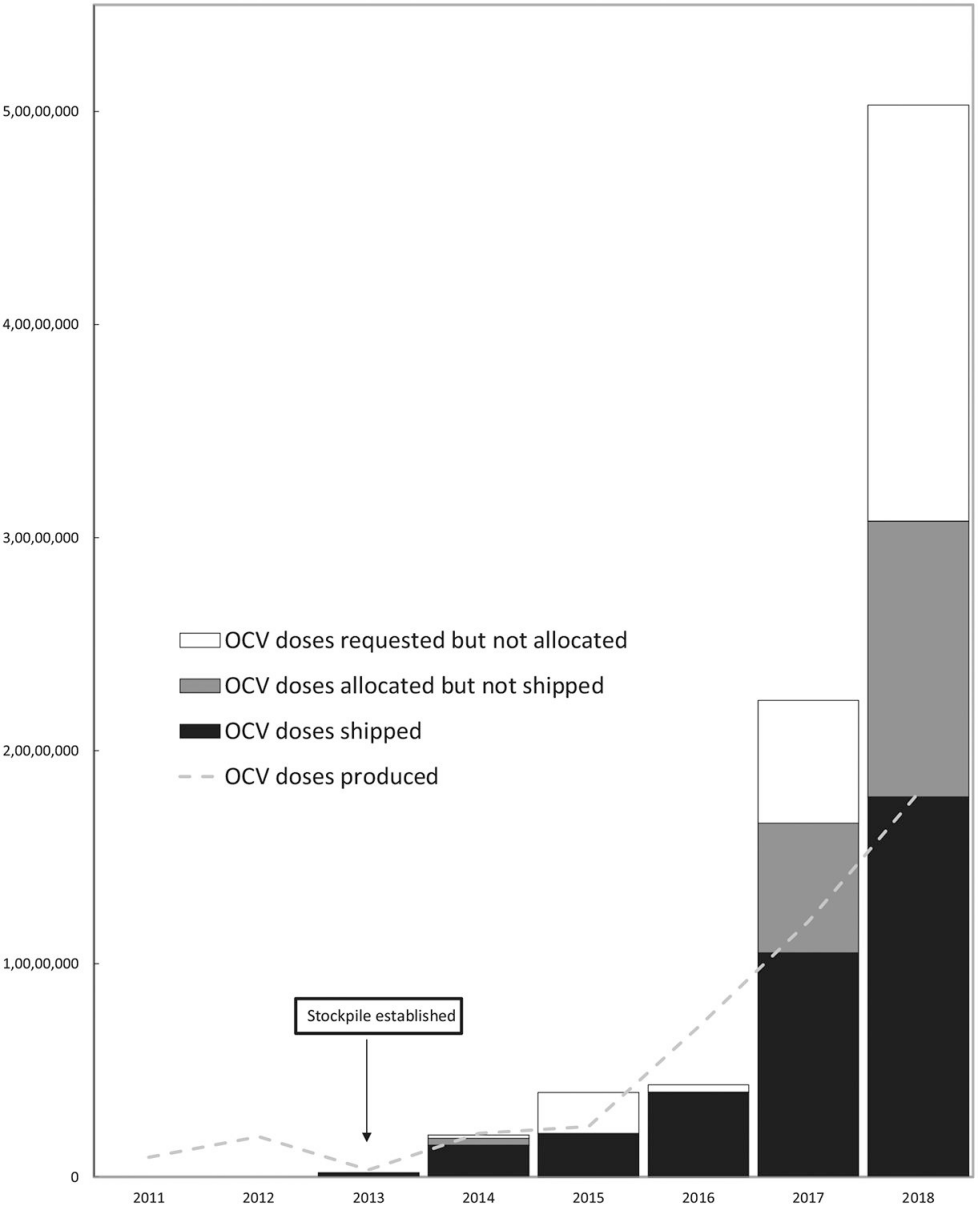
Global oral cholera vaccine use, demand, and production, 1997–2018. Legend to Fig. 1: in 2018 large multi-stage GTFCC requests were submitted (by DRC, Haiti, Nigeria, Somalia, Sudan, Uganda, and Yemen) for a total of 38.1 million doses, some of which were still in process at the time of writing. If approved these requests result in multiple shipments that may take place across multiple years in function of vaccine availability.

Since its creation in 2013 up until all of 2017, 83,509,941 OCV doses have been requested from the stockpile by ministries of health and partners from 24 countries, of which 55,409,160 (66.3%) doses were approved (either by the ICG or the GTFCC OCV WG) and 36,066,010 (43.2% of requested and 65.1% of approved) shipped within 83 deployments to 22 countries. In three instances countries (in Chad in 2014, in Yemen in 2017 and in DRC in 2018), emergency requests were approved by the ICG, but later canceled by requestors. The number of doses shipped has roughly doubled each year, increasing from approximately 200,000 in 2013, to 1.5 million in 2014, 2 million in 2015, 4 million in 2016, 10.5 million in 2017, and 17.8 in 2018. The proportion of requested doses approved was 100% in 2013, 92.0% in 2014, 54.6%, in 2015, 93.0% in 2016, 73.6% in 2017, and 60.4% in 2018. Virtually all doses approved in 2013–2016 were shipped. However, of the 16.0 million doses approved in 2017, only 10.0 million (62.2%) were shipped, while in 2018 17.5 million of the 30.4 million approved (57.4%) were shipped. In total 19.3 million of the 55.4 million doses approved (34.9%), remained to be sent due to insufficient supply (Fig. 1).

Thirteen requests for a total of 3.4 million doses, were not approved. They were all processed through the ICG framework. Five were for outbreak response (three were not approved because the epidemics were considered “too mature” for OCV to have an impact, one because the risk of spread was assessed to be low, and one because the vaccination strategy and the target group represented only by children were not considered appropriate), six for humanitarian crisis (mostly because the risk of cholera was deemed to be low and not immediate; in fact two of these requests were advised to resubmit through the GTFCC as part of plans for controlling endemic cholera), and two for endemic use (one came in 2015 when the GTFCC framework was not yet established and was not approved since priority was given to emergency requests and the other in 2016, which was redirected as part of a larger request to the GTFCC framework and eventually approved).

**Fig. 2.**
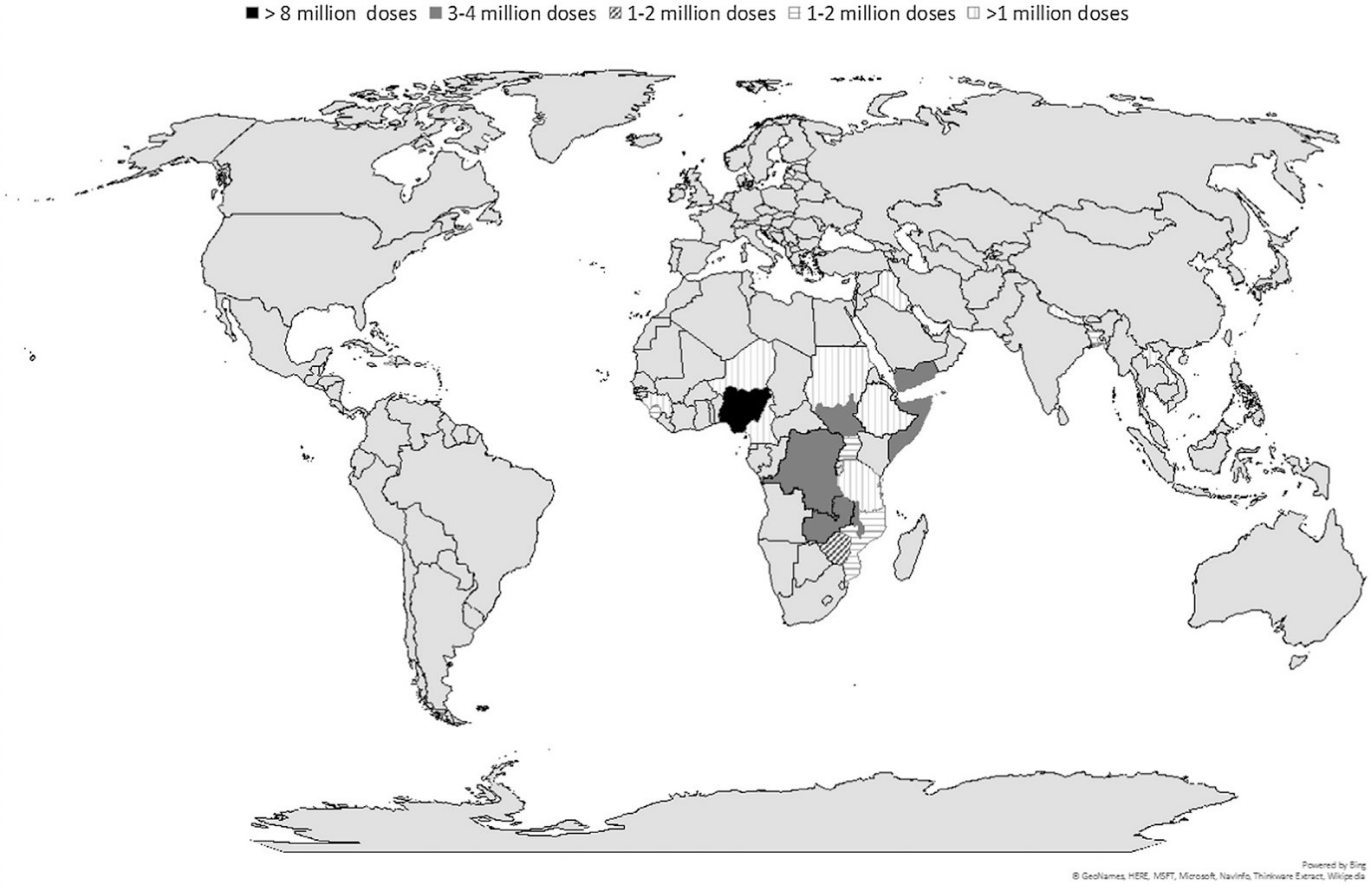
Countries (n = 22) receiving OCV from the stockpile, 2013–2018.

The majority (73.5%; 61/83) of deployments were in the African Region. The three countries receiving the most doses were Nigeria (8.1 million), South Sudan (3.7 million), and Zambia (3.6 million) (Fig. 2). In 2015, 200,000 doses were shipped to Bangladesh for a clinical study and were therefore excluded from the statistics presented in this paper. The number of countries using OCV from the stockpile has increased over the years from one (Haiti) in 2013, to six in 2014, seven in 2015, eight in 2016, nine in 2017, and 11 in 2018.

**Fig. 3.**
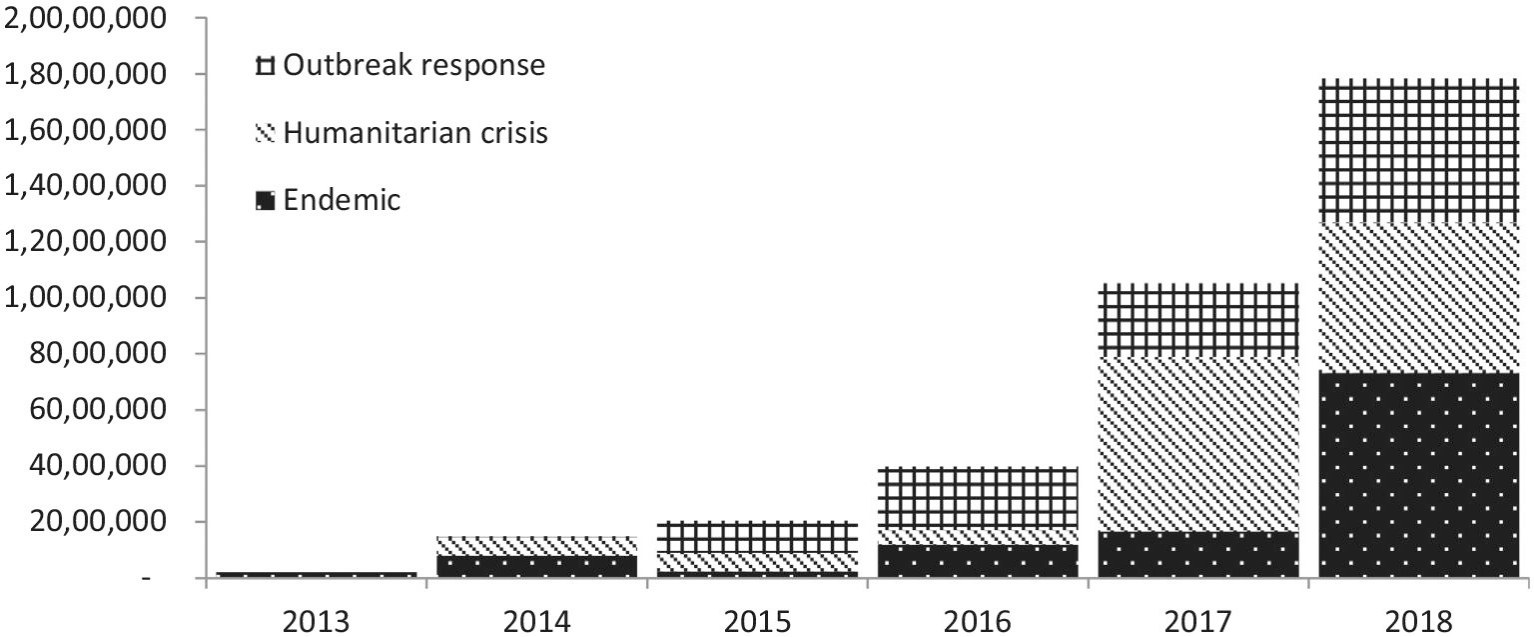
Oral cholera vaccine doses shipped by setting and by year since the creation of the stockpile, 2013–2018.

Approximately 24.7 million doses (68.4%) were shipped to emergency settings, of which 13.5 million (54.3%) were deployed during humanitarian crises and 11.2 million (45.5%) for outbreak responses; while 11.4 million (31.6%) were shipped for the purpose of contributing to controlling endemic cholera in hotspots (Figs. 2 and 3).

**Fig. 4.**
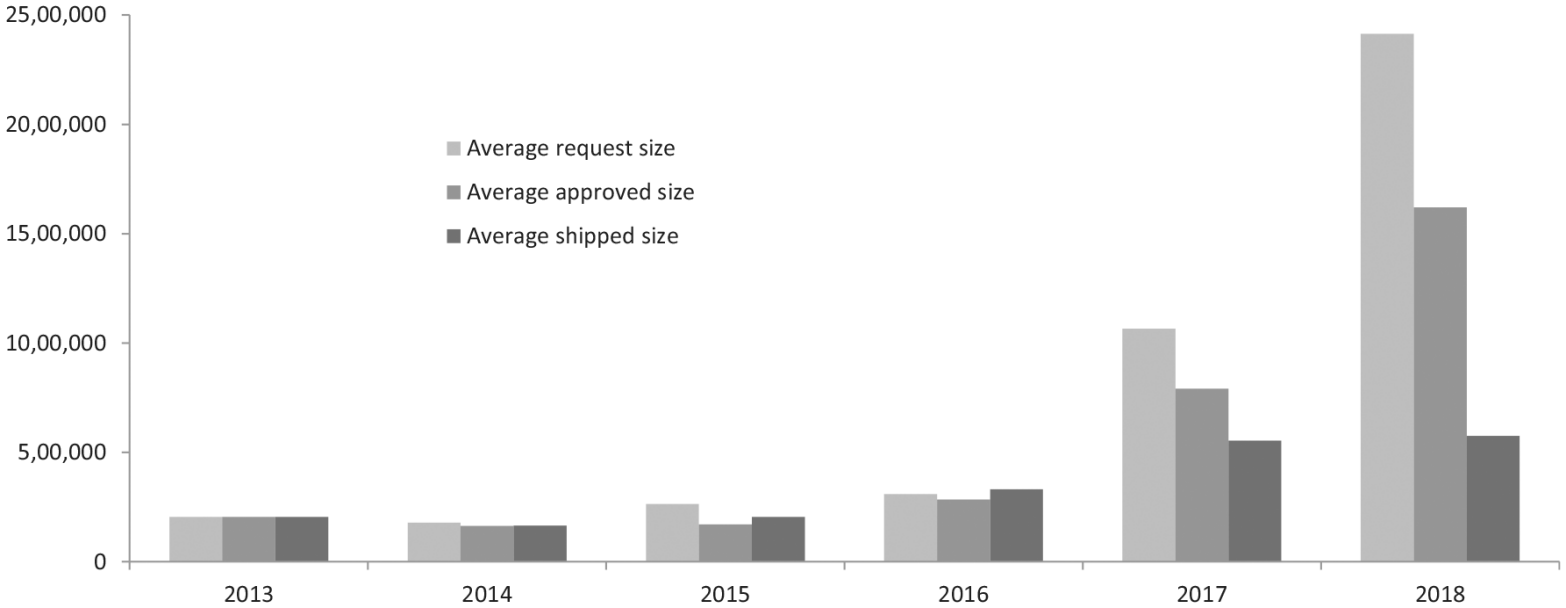
Average number of oral cholera vaccine doses requested from stockpile per request, approved, and shipped, by year, 2013–2018.

For each request, the average number of doses requested was 1.0 million, with an increasing trend from 0.20 million in 2013 (one request), 0.18 in 2014 (11 requests), 0.26 million in 2015 (15 requests), 0.31 in 2016 (14 requests), 1.07 million in 2017 (28 requests) to 2.4 million in 2018 (41 requests). This resulted in 0.71 million doses approved on average and 0.44 million doses shipped on average per request, with a comparable increasing trend from 2014 to 2018. The average proportion of doses approved out of requested from 2013 to 2018 was 100.0%, 92.0%, 64.5%, 91.8%, 74.2%, 67.1% (Fig. 4).

### 3.2. Campaigns

The 83 deployments resulted in 104 campaigns. Not all campaigns had reported the results at the time of writing, but so far 19,300,891 people were targeted during a first round, of which 17,417,707 (90.3%) were vaccinated; while 14,840,677 people were targeted with a second dose, of which 13,282,965 (88.2%) were vaccinated.

The average number of campaigns conducted per country was 4.7, with South Sudan conducting 37 campaigns, Malawi 14, Haiti 13, Nigeria 10, and the rest of the countries between one and three campaigns.

The most common vaccine delivery strategy was fixed post, followed by a combination of mobile and fixed strategies; on two campaigns, both targeting the fishermen communities living on Lake Chilwa in Malawi, self-administration was piloted for the second dose [26-28] (Table 1). All campaigns were planned according to a two-dose vaccination schedule, except one campaign in Juba, South Sudan, in 2015, where a single dose approach was used for outbreak response [29].

The median duration of a campaign was five days for both rounds. The interval between first and second round ranged from 5 to 234 days with a median of 16 days. The campaigns with the longest interval between rounds were in Lusaka, Zambia, in 2016 with 234 days, in Kalemie, DRC, in 2013–2014 (note that doses requested from the stockpile were for the second round only) with 215 days, and in Sud and Grande Anse, Haiti in 2016 with 189 days. Haiti and Zambia administered a first dose to cover the highest population number in an emergency setting and delivered the second dose when more vaccine became available, several months later. The long delay in Kalemie was due to insecurity resulting in operational and access constraints.

Administrative vaccination coverage of the first round ranged from 45.0% to 128.3%, with an average of 92.1%; while for the second round the range was 42.7–140%% and the average 88.2% (Table 2). Vaccination coverage surveys were documented following at least 31 campaigns. The estimated two-dose coverage ranged from 27.5 to 95.3%, with an average of 69.9%; while the estimated coverage with at least one dose ranged from 67.0 to 98.7%, with an average of 84.6% (Table 2).

The most common reason for non-vaccination was absence during the campaign (e.g. conflict with working hours); other reasons included lack of awareness regarding the occurrence of the vaccination campaign, being too busy to get the vaccine or believing that the vaccine was unsafe or ineffective [17,26,30,31]. Another reason cited for non-vaccination during door-to-door campaigns was that the teams were not visiting the respondents’ residential structures [17]. Vaccine shortage was documented at least once [26]. One study conducted in South Sudan pointed to heightened fears of insecurity and disease, contributing to the community’s perception of cholera as a serious illness and increased trust in United Nations and NGOs providing the vaccine to IDPs, as reasons for accepting OCV [30].

No serious AEFI was reported in any of the campaigns. Minor AEFIs were reported, including mainly minor gastrointestinal symptoms, such as nausea, abdominal pain, vomiting, diarrhea and fever [16,17,26,32]. There was one documented occurrence of rash following vaccination [17]. One study followed a cohort of pregnant women after the campaign in Nsanje, Malawi, in 2015 [33] and found no increased risk of complications in vaccine recipients [30].

Data on costs were available from six campaigns from 2013 to 2016 [34,35]. The cost of vaccine was constant at 1.85USD per dose. The cost of international shipment ranged from 0.07 to 0.35 USD per dose, with a mean of 0.16 USD. The operational costs of vaccination in the field ranged from 0.41 to 2 USD per dose with a mean of 0.93 USD per dose. In total, the cost of administering one dose of vaccine ranged from 2.33 USD to 3.97 USD, with a mean of 2.98 USD.

In emergency situations, the median time from the event (i.e. first laboratory confirmation of cholera or occurrence of humanitarian emergency) to the receipt of the official OCV request was 26 days (range 12–206 days). It took a median of five days (range 0–180 days) from receipt to request approval. This includes the time required for countries to provide additional information, when requested. The median time from approval to arrival of vaccines in the country was 13 days (range, 4–24 days) and the median time from arrival to the start of vaccination was 15 days (range, −2 to 87 days – the negative count indicates that the country may start to vaccinate with doses available from previous campaigns). In total, the sum of median times from the occurrence of the event to one week after the end of the first round (time required for immunity to develop) was 66 days.

## 4. Discussion

Since the OCV stockpile creation in 2013, with 104 campaigns conducted in 24 countries using more than 36 million doses, the stockpile allowed for a significant increase in OCV use in cholera-affected countries. OCV was well accepted (as indicated by generally high coverage) by the population and its use demonstrated to be safe and feasible for both emergency response and endemic cholera control. In general, vaccination was an inexpensive and timely intervention, although timeliness was a limiting factor in case of emergency campaigns conducted for outbreak response. The experience described so far demonstrates that countries are increasingly integrating OCV use in their cholera control strategies generating further demand, which results in significant growth in vaccine supply.

The first explanation of the increased OCV use since the creation of the stockpile is the availability of prequalified vaccines. From 2011 by the end of 2018, through all of 2018, 44 million doses of OCV had been produced by the two manufacturers, from approximately two million doses per year between 2013 and 2014, to seven million in 2016, 12 million in 2017 and 18 million in 2018. This is is in stark contrast compared to the previous 15 years (1997–2012) with 13 campaigns implemented and 1.4 million doses used [11,23]. Another likely reason for increased demand of OCV globally is its use by more and more countries adding to the shared experience and knowledge gained and stimulating the global community to consider this tool.

Although the use of OCV in non-emergency situations (i.e. endemic use) has been steadily increasing due to increased supply, most requests, so far, have been for use in emergencies. Hence, not surprisingly the African Region, which is currently the most affected by cholera outbreaks, remains the region with the highest number of OCV deployments. OCV use in the Eastern Mediterranean Region has mostly been related to emergency situations as well, in the context of the humanitarian crises, whereas its use in the American region was localized in Haiti, mostly for endemic cholera control. Finally, and perhaps counterintuitively, the region with the lowest OCV use was South East Asia. Cholera outbreaks tend to go unreported in this region [36], making it less likely for them to request OCV in emergency. With increased availability of OCV for non-emergency use, this situation could change soon, and larger requests may be expected globally. In fact, starting from 2017 several countries (DRC, Haiti, Malawi, Nigeria, South Sudan, Uganda, Yemen, and Zambia) submitted large requests to the GTFCC OCV WG and received approval to conduct multiple campaigns with the administration of millions of doses over longer periods of time, as part of multisectoral NCPs. However, while vaccine production has increased, allowing for these larger requests, OCV supply remains still constrained, resulting in the gap observed since 2017 between the average numbers of doses approved and the number of doses ultimately shipped.

The campaigns using stockpiled OCV have confirmed that OCVs are safe, as seen in the clinical trials [37-42] and in the early vaccination campaigns conducted before the establishment of the stockpile [43-51]. One observational cohort study in pregnant women was conducted following the deployment of OCV in Malawi in 2015 [33], and has contributed further evidence of the safety of OCV in pregnant women [52], previously only measured in retrospective studies [53-55], leading to the WHO recommendation to include this group in the target population [9]. Furthermore, the generally high coverage observed during all the campaigns confirms that OCV is an intervention which is well accepted [16,30,56], with some exceptions related to the relative lower coverage sometimes observed in adult males, who are not traditionally the target for vaccination and are more likely to be at work during vaccination; and some decrease in coverage during second rounds due probably to the misunderstanding by the population that the vaccine is given with only one dose [47,57,58,49].

Although there was significant fluctuation in delivery costs depending on the settings, OCV was confirmed to be generally a low-cost intervention, in line with pre-existing studies [59-61]. However, documenting costs is only the first step in economic analysis and more analysis of cost-effectiveness data is needed [62-64].

This review also demonstrates that OCV is an easy intervention which can even be self-administered, resulting in a reduction of the delivery costs [26], the main limitation being the cold chain requirements [43,48,57,58,65]. OCVs have demonstrated good heat stability [66,67]. While Shanchol has already been approved for use in a controlled temperature chain (CTC), efforts are ongoing to grant label variation to allow for its at temperatures of up to 40 °C for all pre-qualified OCVs, similar to the meningitis A and human papillomavirus vaccines [68,69]. This will facilitate considerably vaccination campaigns in the field. In addition since 2018 Euvichol is now presented in plastic tubes instead of glass vials which has further facilitated delivery in the field and administration.

Although OCV use so far has been timely at the delivery stage with campaigns often lasting less than one week, achieving good timeliness is challenging for emergency use. A median delay of 3 months between the occurrence of an emergency and the start of the first round is unsatisfactory. Late outbreak confirmation, due to poor laboratory capacity or reluctance of countries to report cholera, further increased this delay. One other factor to consider was the delay between first dose and second dose. Supply constraints also play a role in delaying campaigns especially when there are not enough doses in stock to allow translating into campaigns all approved campaigns, making further prioritization necessary.

From a response point of view, the experiences with OCV demonstrated that two OCV doses provide protection against cholera for at least three years, and that one dose provides at least short-term protection [12]. However, although OCV also seems to provide herd protection [70-75], evidence with regards to its impact in reducing the cholera risk at a population level and changing the course of outbreaks is mostly theoretical [76-79], with only a few controlled studies being reported [74,80-82], and could be coincidental.

Another challenge which is directly connected to the impact of the vaccination, is the implementation of OCV simultaneously with other preventive interventions, especially the strengthening of WASH [19]. This can be explained in part because of the difficulties in rapidly improving access to WASH in settings where OCV is implemented, especially in emergencies [16,17,82-84]. Current efforts driven by the GTFCC include the promotion of OCV use within integrated multisectoral cholera control plans [85-87] as an essential requirement for accessing OCV for endemic use.

This review is subject to a number of limitations. First, general information on OCV campaigns was not standardized or recorded systematically in a way that could be easily analyzed. This applies less to the data on requests, which are handled by a central location since both ICG and GTFCC secretariats are housed within WHO headquarters. However, it affects campaign data, the quality of which is dependent on the local context and on the implementing agency’s reporting capacity. Systems to allow systematic and timely reporting (campaign implementation reporting, costs, coverage survey results, other monitoring and evaluation activities, etc.) should be put in place as a requirement to access OCV. A second limitation relates to the availability of systematic data on cholera disease in the countries requesting and using OCV. Although this limitation doesn’t directly affect the quality of OCV utilization data, having reliable and systematic surveillance data allows to better plan OCV campaigns (and all other cholera control interventions) and evaluate their performance.

In conclusion, since the creation of the stockpile, increased availability and demand from countries have contributed to a cycle of increased supply and increased use. The stockpile has also confirmed its role as resource for operational research to inform vaccination strategies locally and globally. This is reflected not only in the increased number of countries using OCV each year, but also in the average size of approved requests, which went from a few hundred thousand doses in 2013 to an average number of doses approved of more than one million per request; demonstrating that the increased availability results in a larger use by countries, motivating other countries to also do the same. On a less positive note, the demand is always exceeding the demand and countries with approved requests are often asked to split their approved requests into smaller shipments and often delay non-urgent campaigns to when supply will allow doses to be shipped.

Further efforts should be directed to ensure that the increased demand, when technically appropriate and realistic, is met with increased supply, especially if the vaccine is expected to be used more and more to control endemic cholera and thus contribute to cholera elimination as laid out by “Ending Cholera: A Global Roadmap to 2030”, the global cholera elimination strategy launched by the GTFCC in 2017. This issue will become even more urgent if countries in the SEARO region will start using OCV systematically like the AFRO region. It is also important to ensure that that OCV is not used alone, but as part of comprehensive package of multisectoral interventions, including provision of adequate, affordable and sustainable safe water supply and sanitation to vulnerable groups, active social mobilization, and reinforcement of surveillance and case management, coordinated at the highest political level within an NCP. Additionally, effort should be allocated to the improvement of the timeliness of response, delivery costs, and more globally in designing innovative and effective strategies for OCV delivery in the different contexts (e.g. balancing “reactive use” in emergencies as quickly as possible with more strategically planned “endemic use” in hotspots). To achieve this, adequate monitoring capacity should be in place to continuously document and refine OCV’s role for global cholera control every time that it is used. In this sense, the momentum generated by OCV campaigns and the mobilization of operational costs should be capitalized to reinforce health systems in general.

## Figures and Tables

**Table 1:** Strategies of vaccine delivery used by oral cholera vaccination campaigns from the stockpile (n = 104), 2013–2018.

Strategy	Campaigns	Percentage
Fixed	35	33.7
Mixed (Fixed; Mobile)	41	39.4
Not specified	15	14.4
Mobile	9	8.7
Mixed (Fixed; Community-based; Self administration)	2	1.9
Mixed (Fixed; Mobile; Road Side)	2	1.9

**Table 2:** Oral cholera vaccination coverage (administrative and estimated) for campaigns by context and strategy of vaccine delivery used, from stockpile, 2013–2018.

	Average of admin coverage first round	Average of admin coverage second round	Average estimated coverage at least one dose*	Average estimated coverage two doses*
Context				
Endemic	93.6%	92.4%	85.1%	69.3%
Humanitarian crisis	82.9%	82.0%	73.2%	68.4%
Outbreak	98.8%	93.0%	86.3%	73.6%
Strategy				
Fixed	86.5%	81.2%	74.5%	62.0%
Mixed (Fixed; Community-based; Self administration)	117.9%	77.9%	87.4%	72.4%
Mixed (Fixed; Mobile)	94.9%	94.3%	87.4%	76.6%
Mixed (Fixed; Mobile; Road Side)	98.3%	98.5%	89.5%	85.1%
Mobile	89.3%	88.5%	74.4%	50.4%
Overall	90.3%	88.2%	84.6%	69.9%

* Based on 31 OCV campaigns reporting coverage survey results.

## Appendix

^1^ Members of the Global Task Force on Cholera Control (GTFCC) Oral Cholera Vaccine (OCV) Working Group: • Agence de Médecine Préventive: Philippe Cavailler, Martin Mengel; • The Bill and Melinda Gates Foundation: Helen Matzger, Tina Lorenson; • Dipika Sur (independent); • Epicentre: Francisco Luquero, Rebecca Grais; • Gavi: Melissa Ko, Adam Soble; • GHESKIO: Vanessa Rouzier, Jean William Pape; • Harvard University: Caroline Buckee; • icddr,b: Firdausi Qadri; • International Federation of Red Cross and Red Crescent Societies (IFRC): Frank Mahoney; • International Medical Corps (IMC): Jill John Kall; • International Rescue Committee: Justine Landegger, Michelle Gayer; • International Vaccine Institute: Julia Lynch; • Johns Hopkins University: Andrew S. Azman, David Sack; • Médecins Sans Frontières: Myriam Henkens, Iza Ciglenecki; • National Institutes of Health: Robert Hall; • Massachusetts General Hospital Center for Global Health: Louise C. Ivers; • Save the Children: Emma Diggle; • Swiss Tropical and Public Health Institute: Mitchell Weiss; • Task Force for Global Health: Alan Hinman; • UNHCR: Kahindo Maina; UNICEF: Imran Mirza (Program Division), Heather Papowitz (Program Division), Guillermo Gimeno (Supply Division), Monica Ramos (WASH); • University of Maryland: Myron M. Levine; • US Centers for Disease Control and Prevention: Kashmira Date,Nandini Sreenivasan* (Disclaimer: Kashmira Date and Nandini Sreenivasan are employees of the US Centers for Disease Control and Prevention. The findings and conclusions in this report are those of the authors and do not necessarily represent the official position of the US Centers for Disease Control and Prevention.)
